# Synaesthetic Colour in the Brain: Beyond Colour Areas. A Functional Magnetic Resonance Imaging Study of Synaesthetes and Matched Controls

**DOI:** 10.1371/journal.pone.0012074

**Published:** 2010-08-10

**Authors:** Tessa M. van Leeuwen, Karl Magnus Petersson, Peter Hagoort

**Affiliations:** 1 Centre for Cognitive Neuroimaging, Donders Institute for Brain, Cognition and Behaviour, Radboud University Nijmegen, Nijmegen, The Netherlands; 2 Max Planck Institute for Psycholinguistics, Nijmegen, The Netherlands; University of Regensburg, Germany

## Abstract

**Background:**

In synaesthesia, sensations in a particular modality cause additional experiences in a second, unstimulated modality (e.g., letters elicit colour). Understanding how synaesthesia is mediated in the brain can help to understand normal processes of perceptual awareness and multisensory integration. In several neuroimaging studies, enhanced brain activity for grapheme-colour synaesthesia has been found in ventral-occipital areas that are also involved in real colour processing. Our question was whether the neural correlates of synaesthetically induced colour and real colour experience are truly shared.

**Methodology/Principal Findings:**

First, in a free viewing functional magnetic resonance imaging (fMRI) experiment, we located main effects of synaesthesia in left superior parietal lobule and in colour related areas. In the left superior parietal lobe, individual differences between synaesthetes (projector-associator distinction) also influenced brain activity, confirming the importance of the left superior parietal lobe for synaesthesia. Next, we applied a repetition suppression paradigm in fMRI, in which a decrease in the BOLD (blood-oxygenated-level-dependent) response is generally observed for repeated stimuli. We hypothesized that synaesthetically induced colours would lead to a reduction in BOLD response for subsequently presented real colours, if the neural correlates were overlapping. We did find BOLD suppression effects induced by synaesthesia, but not within the colour areas.

**Conclusions/Significance:**

Because synaesthetically induced colours were not able to suppress BOLD effects for real colour, we conclude that the neural correlates of synaesthetic colour experience and real colour experience are not fully shared. We propose that synaesthetic colour experiences are mediated by higher-order visual pathways that lie beyond the scope of classical, ventral-occipital visual areas. Feedback from these areas, in which the left parietal cortex is likely to play an important role, may induce V4 activation and the percept of synaesthetic colour.

## Introduction

If you would have digit-colour synaesthesia, every phone number would have its own unique colour-code – very helpful for memorisation. You would not know what it is like to see digits without an associated colour; to you, it would be completely natural to see that 2 is yellow and 3 is green, and it would have been that way for as long as you could have remembered [1]. Moreover, you would not be able to ignore the colours, or not see them. The colours would always be there, and always remain the same [1]. In people with synaesthesia, sensations in one sensory or cognitive modality lead to additional experiences in a second, unstimulated modality. A common form is grapheme-colour synaesthesia, in which letters and/or digits involuntarily [2], [3] elicit a specific, idiosyncratic colour (e.g. A elicits red). In this study, we investigate the neural correlates of grapheme-colour synaesthesia.

Neural explanations for synaesthesia include (local) anatomical hyperconnectivity [4]–[6], as well as disinhibited feedback mechanisms with an associated increase in functional connectivity [7], [8]. In neuroimaging studies, deploying various different methods (e.g. functional magnetic resonance imaging, positron emission tomography, source localisation of electroencephalography data), enhanced brain activity for synaesthesia has been reported in ventral-occipital colour areas [9]–[13] and in left parietal cortex [10], [11], [13]–[15]. Anatomical differences have also been found in these regions [16]–[18]. Recently, Hubbard [19], [20] has put forward a two-stage (or integrated) model of grapheme-colour synaesthesia. In the model, perception of the synaesthetic colour and binding of the colour to the grapheme are modelled as separate processes; for the former, colour areas in fusiform gyrus are deemed crucial, for the latter the parietal cortex. Parietal cortex is involved in spatial feature binding [21], [22] and inhibition of right parietal cortex with transcranial magnetic stimulation can disrupt synaesthesia [23], [24].

In spite of the neuroimaging findings and existing theories, the exact neuronal mechanisms underlying synaesthesia are still not well understood. The neural correlates of synaesthesia are of interest because people with synaesthesia experience sensations without receiving direct sensory input to warrant these sensations; still, their experience is very real and salient. Synaesthesia can provide insight into mechanisms of perceptual awareness, and inform us about how sensory inputs are combined in our brain. Here, our aim was to find out whether the experience of synaesthetically induced colour recruits *exactly* the same colour sensitive regions in the brain as real colour experience. Although synaesthesia related activity has been reported in colour processing areas [9], [10], we explicitly tested whether synaesthetic colours can *affect* real colour processing in the brain. This would imply truly shared neural machinery. The outcomes would inform us about the level of visual processing at which colour experience induced by synaesthesia is mediated.

Behavioural studies show that synaesthetic colour experiences can influence the reaction times of judgments on real colour [7], [25], [26]. Synaesthetically induced colour also resembles real colour perception in a wide range of perceptual tasks, for example perceptual crowding, visual grouping and visual search, apparent motion, and the watercolour effect [4], [27]–[29]. Synaesthetic colours may even adhere to early visual colour-opponency mechanisms [30]. In a study by Nicolić, Lichti, and Singer [31], incongruent Stroop colours were chosen to be maximally opponent to the colours that were induced by synaesthesia; larger interference effects were found for opponent colours than for non-opponent incongruent colours.

Together these findings suggest that synaesthetic colour perception shares at least several neural processing steps with real colour perception, possibly also in early visual areas. On the other hand, synaesthetes subjectively report that synaesthetic and real colours do not mix [32], and that synaesthetically induced colours are difficult to express in real colour terms. Similarly, perceptual resemblance to real colour has not always been reproduced (e.g., in visual search, [33], [34]). Hong and Blake [35] have shown that synaesthetic colours are not influenced by brightness contrast, do not induce hue cancellation, and do not affect real colour perception in equilibrium yellow settings. The authors therefore argued that the earliest neural correlates of synaesthetic colour perception must lie beyond primary visual cortex, an issue that we investigated further in the current study.

Our experiment was conducted on the basis of a previous study (van Leeuwen, Petersson, Langner, Rijpkema, and Hagoort, submitted) in which we demonstrated that the repetition of real colour induces repetition suppression effects in functional magnetic resonance imaging (fMRI): we found a decrease in the blood-oxygenated-level-dependent (BOLD) response for repeated stimuli [36], [37]. Repetition suppression (RS) can be regarded as a priming effect in the brain, and occurs when there is sufficient overlap in neuronal processing between the first (prime) and the second (target) stimulus. Given this assumption, RS is a sensitive method that allows for precise localisation of the representation of specific stimulus features. Please note that repetition suppression for stimulus features in sensory cortex can be completely independent from observable measures like reaction times [38]. We have shown RS effects for real colour in visual area V4α (van Leeuwen, Petersson, Langner, Rijpkema, and Hagoort, submitted), in which colour processing is already past the first, colour-opponent stages [39]. V4α can be regarded as a higher-order colour processing area. For the repetition of real colour, we found stronger RS effects for the condition in which the colour of the target square was congruent with the colour of the prime, than for the condition in which the prime and target colours were incongruent. In the current experiment (including the same subjects as the real colour study) we used graphemes as primes instead of coloured squares. The graphemes elicited a vivid synaesthetic colour for the synaesthetes, which could either be the same (congruent) or different (incongruent) from the physical colour of the subsequent target square. We predicted more RS effects for the congruent colour condition than for the incongruent colour condition in those brain areas where the neuronal processing of synaesthetically induced colours and real colours is (partly) shared. We compared the neural correlates of synaesthesia-colour priming to the effects obtained for real colour, to see whether synaesthetically induced colour led to similar RS effects as real colour.

To optimise our sensitivity we included a large cohort of synaesthetes (N = 21) and matched controls. Apart from the congruent synaesthetic colour (CC) and incongruent synaesthetic colour (IC) conditions, a control condition was included in which the primes did not elicit a colour at all (non-inducing condition (NC)). No repetition suppression was predicted for NC trials, because in colour sensitive regions [40], [41] the responses to achromatic stimuli (all stimuli were presented in black) are typically much weaker than responses to chromatic stimuli. The control participants did not experience synaesthesia for any of the prime stimuli; hence for the controls we did not predict any modulation of the BOLD response due to the manipulations in the primes. Participants indicated the colour of the target square with a button press, and reaction times were measured to compare the effects to previous behavioural findings for synaesthetic priming [25], [26].

We specifically predicted RS effects in ventral-occipital parts of the brain, in which grapheme and colour processing areas are located in close proximity [4]. To help with the interpretation of our effects, we performed a functional localiser experiment (Experiment 1) in which we mapped grapheme and colour sensitive areas, and localised the main effects of synaesthesia. Graphemes that were inducing vivid synaesthetic colours were contrasted against non-inducing control graphemes to localise synaesthesia related activity (stimuli from both conditions were presented in black). False font stimuli (also presented in black), which resembled well known graphemes in visual complexity but did not have any meaning, were compared to the non-inducing control graphemes to help identify the neural correlates of grapheme processing. To map colour responses, we included a condition in which the non-inducing control stimuli were presented in colour instead of black. We also compared synaesthetic colour experience to real colour experience to capture the added quality of synaesthesia. The resulting activation patterns for graphemes and colours were used as volumes of interest (VOI) to restrict our search for colour-specific RS effects in the synaesthetic priming experiment (Experiment 2).

Synaesthesia also has a spatial component: *projector* synaesthetes report seeing the colours ‘on’ the grapheme, while *associators* do not and experience the colours in their ‘mind's eye’ [32]. In both Experiment 1 and 2, we investigated the influence of the synaesthetes' projector-associator status on synaesthesia related brain activity: these individual differences can influence experimental results [10], [17], [32].

## Materials and Methods: Experiment 1

### Localising Synaesthesia, Grapheme, and Colour Areas

In Experiment 1 we identified grapheme areas, colour areas and synaesthesia-related areas in the brain, and investigated the effect of projector-associator status on synaesthesia-related activity.

### Participants

#### Synaesthetes

Twenty-one synaesthetes aged 18–37 (mean age 26 years, *SD* = 4.9 years, 2 men, 2 left-handed, 1 ambidextrous) participated. Selection was performed on the basis of a questionnaire that assessed synaesthetic experiences, medical history, and handedness (self-reported hand preference). From the general part of the questionnaire (30 questions on synaesthesia, comprising questions like “How long have you experienced synaesthesia?” and “Did the experience change over time?”), it was determined whether the participants fitted the profile for developmental synaesthesia. All synaesthetes experienced grapheme-colour synaesthesia since early childhood; 20 reported additional synaesthesias, for example time units inducing colour (n = 18) and/or shapes (n = 15), and sound-colour synaesthesias (n = 8).

In the questionnaire synaesthetes reported the colour and intensity of their synaesthesia for 26 letters of the alphabet, digits 0–9, 15 familiar non-alphanumeric symbols (e.g. #, %) and 13 ‘false font’ stimuli (unfamiliar symbols derived from Cyrillic, Greek, and Arabic, not resembling Latin letters or numbers in shape). We tested the consistency of the synaesthetic experiences over time to verify genuine synaesthesia [2], [42]. A surprise re-test on 20 graphemes, taking place by phone 8–13 months (mean 11.0 months) after the initial study yielded an average consistency score of 91% (*SD* = 7.5%), similar to previously reported consistency scores (e.g., [25], [42]).

We characterised the synaesthetes on the basis of the spatial location of their colour experiences [21], [32]. As classification criteria we used the participants' detailed descriptions of the appearance of their synaesthesia, in which we explicitly asked them to describe the spatial location of their experiences. For clarity, we added 9 specific questions on the location and shape of the synaesthetic colours. Synaesthetes indicated how much they agreed (on a 5-point scale) to sentences that fitted either best with a projector, mental screen projector, or an associator viewpoint (similar to the procedure in [17]). The scores on this scale determined how they were characterised. Seven synaesthetes were classified as ‘projectors’, who experience the colour as an overlay projection on the graphemes themselves; 8 as ‘mental screen projectors’, whom experience the colours in external space but not on the graphemes (in some papers, these synaesthetes are referred to as associators, e.g. Ward et al. [21]); and 6 as ‘associators’, who experience synaesthesia as a strong association between the grapheme and the colour.

#### Controls

Nineteen control participants aged 19–38 (mean age 26 years, SD = 4.7 years) who did not report synaesthesia were individually matched to the synaesthetes on sex, age (±3 years), handedness, and educational level. Mean ages did not differ between the groups: *t*(18) = −1.46, n.s. Controls completed a pre-screening questionnaire to assess their medical history and handedness and were asked to associate a colour with 20 graphemes. Unannounced re-testing of the colour associations after 5–9 months (mean 6.6 months) yielded a consistency score of 32% (*SD* = 18%), which was significantly lower than the synaesthetes' score; *t*(38) = 13.4, *P*<.001.

All participants had normal or corrected to normal vision, reported no colour blindness and were able to discriminate the experimental colours. None reported a neurological or psychiatric disease. One participant was excluded prior to analysis after reassessment of her medical history, leaving 20 synaesthetes. Written informed consent was obtained from all participants prior to scanning and the study was approved by the local ethics committee of the Radboud University Nijmegen, in accordance with the Declaration of Helsinki.

### Materials

Upon arrival each synaesthete indicated (with Microsoft Powerpoint) the synaesthetic colours for 10 customised graphemes selected from the questionnaire. Eight synaesthesia-inducing graphemes for which the chosen colours matched well to the experienced synaesthesia, and that elicited vivid colours, were chosen for the *synaesthesia* condition. For the *non-inducing control* condition we selected 8 graphemes that elicited no synaesthesia (as indicated in the questionnaire). For 14 synaesthetes, several (3.6 (*SD* = 1.4) on average) familiar non-alphanumeric symbols (e.g. #, %) were included in the non-inducing control condition because there were not enough non-inducing alphanumeric graphemes. Stimuli from the synaesthesia and the non-inducing conditions were presented in black. To create the *colour* condition, the non-inducing graphemes were displayed in random colours unrelated to synaesthetic experiences. Finally, 8 non-inducing, unfamiliar symbols with a visual complexity comparable to regular alphanumeric characters were chosen to constitute the *false font* condition (also presented in black). These symbols did not have any meaning, in contrast to frequently used alphanumeric symbols. For one grapheme-gender synaesthete, genders of the stimuli were divided equally across experimental conditions.

### Stimulus Presentation

Stimuli were presented against a light grey (full screen, 9.1 cd/m^2^) background, using Presentation (version 10.2, *Neurobehavioral Systems Inc*., www.neurobs.com). Non-colour stimuli were presented in black to ensure high contrast with the background, which may influence the strength of synaesthetic experiences [43]. Bright, distinct colours were used for the colour condition (not luminance-matched to the other conditions). All alphanumeric graphemes were 2.0° tall while non-alphanumeric symbols ranged from 1.3°–2.7° tall. Stimuli were presented in the centre of a 44.5×33.5 cm display screen in the scanner tunnel, placed at a viewing distance of 60 cm (controlled by a Dell Pentium IV Windows XP computer, display mode 800×600 pixels, 60 Hz, projected by a EIKI X986 beamer).

### Procedure

Participants passively viewed pseudorandom blocks of A) 8 synaesthesia-inducing graphemes; B) 8 non-inducing control graphemes; C) 8 coloured non-inducing graphemes and D) 8 false font stimuli. Stimulus order within the blocks was randomised. Control participants viewed the same stimulus list as the synaesthete to whom they were matched. Each stimulus was presented for 1500 ms with a 500 ms inter-stimulus interval; between blocks, a central black fixation cross was presented for 10 seconds. Six blocks for each condition yielded a total runtime of 11 minutes (350 MR images). In addition to the standard sound-attenuating headphones, two synaesthetes who reported synaesthesia for the scanner sounds wore earplugs.

### Image Acquisition

MR data were acquired with a 3.0 Tesla Siemens TrioTim MR scanner and an 8-channel head array (Invivo). First, a high-resolution T1-weighted structural image was acquired for each participant (MPRAGE, TE = 2.96 ms, TR = 2300 ms, 256 mm FOV, 256×256 matrix, 1 mm^3^ resolution) with an acquisition time of 5 minutes, accelerated with factor 2 by GRAPPA parallel imaging [44]. A single shot gradient echo-planar imaging (EPI) sequence was used to acquire functional MR images (29 slices, TE = 30 ms, TR = 1840 ms, flip angle = 80°, 224 mm FOV, 64×64 matrix, 3.5×3.5 mm voxel size, 3.0 mm slice thickness, 0.5 mm slice gap).

### Data Analysis

MR data were preprocessed and analysed with SPM5 (Wellcome Department of Imaging Neuroscience, www.fil.ion.ucl.ac.uk/spm/software/spm5). Prior to analysis, the first 5 volumes of each subject were discarded to avoid transient T1 effects. To correct for head motion, images of each subject were spatially realigned to the first image using a six parameter rigid body transformation for each image. Slice timing correction was applied and the images were normalised to the standard EPI template of SPM5 to allow for group inference. Finally all images were spatially filtered using a 10 mm FWHM isotropic Gaussian filter.

Statistical analyses were performed on the basis of the General Linear Model (GLM) framework. For each subject the design matrix was constructed and the BOLD signal was modelled by the canonical haemodynamic response function (HRF). A high-pass filter (128 s cut-off) was used to remove low-frequency effects and global scaling was applied to remove various global effects of no interest. The effects of interest were modelled with boxcar responses (synaesthesia, non-inducing, colour, and false font blocks) and included in the design matrix in a blocked design. The six realignment parameters, obtained during preprocessing, were included in the model as covariates of no interest. Parameter estimates were obtained for each condition and each participant to generate relevant contrast images and allow for second-level random effects analysis. Coordinates of peak activity are reported in MNI coordinates in the order (x, y, z) and the initial threshold was *P*<.001_uncorrected_ at the whole brain level, with a cluster-level statistic of *P*<.05_FWEcorrected_. Corresponding brain regions and Brodmann areas were retrieved from the Talairach Daemon database server [45] and verified with the SPM5 Anatomy toolbox [46]. Mean parameter estimates for Region of Interest (ROI) analyses were extracted using MarsBaR [47].

## Results and Discussion: Experiment 1

Nineteen synaesthetes and nineteen matched controls were included in the analysis of Experiment 1. One synaesthete was excluded because this participant was excluded from the analysis of Experiment 2 (see below); we preferred to keep the number of subjects identical across the two experiments.

### Localising Grapheme Areas

**Figure 1:**
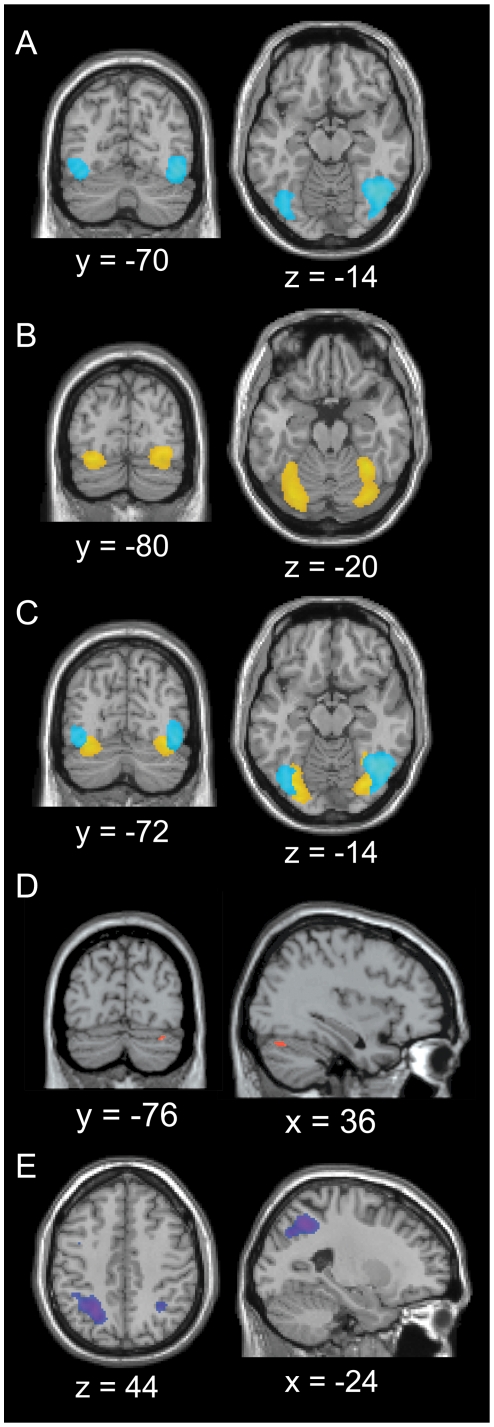
Localising graphemes, colour, and synaesthesia in the brain. fMRI results of localiser Experiment 1. A–C. Coronal (left) and axial (right) slices (N = 38 at whole brain *P*<.05_FWEcorrected_, extent threshold 20 voxels). A. Grapheme areas (false fonts > non-inducing graphemes). B. Colour areas (coloured graphemes > non-inducing graphemes). C. Grapheme (blue) and colour areas (yellow) overlaid. D–E. Effects of synaesthesia, showing positive interactions for synaesthetes (N = 19 synaesthetes, N = 19 controls), at whole brain *P*<.001_uncorrected_, extent threshold 20 voxels. D. Synaesthesia > non-inducing graphemes (red), axial (left) and sagittal (right) slices showing right fusiform gyrus activation. E. Synaesthesia > coloured graphemes (blue-purple), axial (left) and sagittal (right) slices, showing left superior parietal lobe activation. Left is depicted on the left.

Non-inducing control stimuli (graphemes) were contrasted to the false font stimuli to localise grapheme areas. In the absence of interaction effects between the groups (at whole brain *P*<.001_uncorrected_) the data were collapsed across synaesthetes and controls, and thresholded more stringently at whole brain *P*<.05_FWEcorrected_ (N = 38). An effect was found only in the right superior parietal lobe (Table 1), but no clusters were found in ventral-occipital cortex as was hypothesised. Several previous studies have reported enhanced activity for unfamiliar symbols (e.g. Korean letters) compared to letters (or pseudowords compared to words) in visual areas [48]–[51]. We therefore computed the reverse contrast of false fonts compared to non-inducing control graphemes (N = 38, whole brain *P*<.05_FWEcorrected_): we found bilateral clusters of activation in the inferior occipital gyrus (Brodmann areas 18/19) and in the anterior section (BA 37) of the fusiform gyrus (Table 1 and Figure 1A).

**Table 1:** Graphemes, colour, and synaesthesia in the brain.

Contrast	Brain region	k	p	MNI (x,y,z)	T-value
**Non-inducing>False fonts** (***P*** **<.05_FWEcorrected_, N = 38**)	R Supramarginal gyrus/Inf parietal lobule (BA 40)	68	0.001	50, −46, 36	6.15
**False fonts>Non-inducing** (***P*** **<.05_FWEcorrected_, N = 38**)	R Fusiform gyrus (BA 37)	1914	0.000	44, −60, −14	11.06
	R Middle/Inf occipital gyrus (BA 18/19)			38, −84, −4	8.16
	R Fusiform gyrus (BA 37)			34, −48, −22	8.11
	L Inferior occipital gyrus (BA 18/19)	913	0.000	−38, −80, −8	8.30
	L Fusiform/Inf occipital gyrus (BA 19)			−40, −70, −14	7.38
	L Cerebellum/L Fusiform gyrus (BA 37)	60	0.001	−34, −42, −30	5.95
	L Fusiform gyrus (BA 37)			−34, −52, −22	5.53
**Coloured>Non-inducing** (***P*** **<.05_FWEcorrected_, N = 38**)	R Fusiform gyrus (BA 37)	1294	0.000	34, −50, −22	10.11
	R Fusiform gyrus (BA 19)			32, −76, −16	8.66
	R Middle/Inf occipital gyrus (BA 18)			34, −86, −6	7.77
	L Fusiform gyrus (BA 19)	1175	0.000	−30, −76, −16	8.89
	L Fusiform gyrus (BA 37)			−34, −54, −20	7.30
**Interaction Synaesthetes>Controls for Synaesthesia>Non-inducing** (***P*** **<.001_uncorrected,_ SVC**)	R Fusiform gyrus (BA 19)	21	0.052	36, −76, −26	4.45
**Interaction Synaesthetes>Controls for Synaesthesia>Coloured** (***P*** **<.001_uncorrected_**)	L Superior parietal lobule (BA 7)	712	0.001	−24, −58, 46	5.03
	L Superior Occipital gyrus/Precuneus			−22, −70, 38	4.11
	L Inferior parietal lobule (BA 40)			−42, −44, 48	4.02

fMRI results of localiser Experiment 1 (N = 38 (synaesthetes N = 19, controls N = 19), random effects analyses, extent threshold ≥20 voxels, whole brain threshold: see table). Cluster size (k), corrected P-values at cluster-level (p), MNI coordinates of local maxima and T-values are listed. Brodmann areas (BA) are in parentheses. R = right, L = left, Inf = Inferior, SVC = Small Volume Correction.

The effects for false font symbols corresponded to previous findings for written words [48] and letter and symbols [49], [51] that are contrasted against baseline activity. However, our obtained activation pattern did not include the anterior left fusiform gyrus which is proposed to mediate sublexical properties of letters and words [52], [53] and is influenced by word frequency [54] and related to task accuracy [55]. Our results suggest that the increased activity for the false font symbols was largely caused by familiarity effects: although matched in visual complexity, the control graphemes were more frequent in written language. Free viewing conditions and the long stimulus exposure (1500 ms) may have induced additional processing for the unfamiliar symbols. The activation pattern for false fonts compared to control graphemes was used as a volume of interest (VOI) in our subsequent analyses, with the explicit note that we cannot claim that this VOI includes areas involved in processing of graphemes per se; rather, the VOI is capturing visual areas that are especially recruited during the complex and abstract visual analyses that underlie symbol and grapheme processing.

### Localising Colour Areas

To localise colour areas we contrasted coloured graphemes with the (black) non-inducing control graphemes. Again, no interaction effects were found at whole brain *P*<.001_uncorrected_, meaning the effects were similar across both groups of participants, and the data were analysed across all 38 subjects (at whole brain *P*<.05_FWEcorrected_). Note that in this contrast, the effect of the graphemes themselves is cancelled out (identical stimuli in both conditions), leaving only the effect of colour. Bilateral clusters were obtained in the fusiform gyrus, located medially and ventrally from the areas involved in grapheme processing (Table 1 and Figure 1B), although there was partial overlap (Figure 1C). Although the coloured graphemes were not luminance-matched to the black non-inducing graphemes, our results (Table 1) corresponded very well to previous reports on colour sensitive areas [39], [40]. The local maxima in the anterior fusiform gyri (BA 37) were within 5 mm of the reported anatomical location of anterior visual area V4α; the more posterior maxima in fusiform gyrus (BA 19) were within 5 mm of the location of V4 ([39] Table 1). We concluded that the obtained activation pattern for colour (at *P*<.05_FWEcorrected_) could be used as a representative subset of colour sensitive areas (VOI) during further analyses.

### Localising Synaesthesia Areas

To localise the effects of synaesthesia we identified brain regions with a positive interaction for synaesthetes compared to controls, for the contrast of synaesthetic graphemes compared to non-inducing control graphemes. This contrast represents the phenomenal experience of synaesthetic colour for synaesthetes that is additive over the stimulus effects for the controls. Note that any possible perceptual effects of the stimuli are cancelled out in this interaction test, because those effects are expected to be present for both groups. An interaction effect for synaesthetes was present within the ventral-occipital colour VOI (used as Small Volume Correction), located at (36, −76, −26) in the right posterior fusiform gyrus (see Table 1 and Figure 1D). The location of the effect is within the extent of colour area V4 as defined by Bartels and Zeki ([39], Table 1). The results confirm the role of ventral-occipital colour areas in synaesthetic colour experience [9]–[11].

Additionally we compared synaesthetic colour perception (synaesthesia condition) to real colour perception (colour condition) to investigate possible differences between the neural correlates. Here, it is the added synaesthetic quality of the experienced colour that would cause any additional effects for the synaesthesia condition for synaesthetes. A significant (whole brain *P*<.001_uncorrected_) positive interaction for synaesthetes compared to controls was found at (−24, −58, 46) in the left superior parietal lobule (see Table 1 and Figure 1E for details). No effects were found in ventral-occipital areas, also not within the colour VOI. Note that in the interaction test, possible perceptual effects of the stimuli (expected for both groups) are cancelled out. Increased BOLD fMRI effects for synaesthesia in the left parietal cortex were found previously by Weiss et al. [14], while diffusion tensor imaging (DTI) and voxel-based morphometry (VBM) studies have shown increased structural connectivity [17] and increased grey matter density [18] in synaesthetes in this region. The cluster in the superior parietal lobule is located within a 5 mm radius of the main effect of synaesthesia in the fMRI study of Weiss et al. [14].

In our data, the two contrasts involving synaesthesia led to different effects in the brain. The perception of synaesthetic colour led to increased activation in colour area V4. When contrasted against real colour perception, however, the added quality of the synaesthetic colour experience led to more activity in the left superior parietal lobe. Our results support the two-stage or integrated model of Hubbard [19], [20], in which fusiform gyrus (V4) is proposed to underlie the perception of the synaesthetic colour, while parietal cortex is hypothesised to induce a type of ‘hyperbinding’ that binds the colour and the grapheme. In our view, both regions are equally crucial for synaesthetic experience and we therefore prefer the term ‘integrated’ model. The ongoing processes in synaesthetic experience are likely to be based on a dynamic interplay between brain areas, and do not involve two clearly separate processing stages. Our parietal effect can be explained in terms of binding of the synaesthetic colour to the grapheme: in the colour condition, synaesthetes *and* controls needed to bind the physical colour of the non-synaesthetic grapheme to its spatial location [24]. But in the synaesthetic condition, no physical colour was present and hence for controls, no binding was necessary. Synaesthetes however did integrate the synaesthetic colour with the grapheme, leading to increased left parietal cortex activity for synaesthetes. We cannot exclude that the parietal effects we observe are due to other processes than binding, but previous findings concerning spatial feature binding in the parietal lobe make our interpretation very likely [22]–[24].

Importantly, no task requirements were present in our experiment which means no special focus was placed on any particular feature of the graphemes (e.g. shape, physical or synaesthetic colour). Cohen Kadosh et al. [56] have shown that task demands can influence fMRI effects of synaesthesia, an insight which can help to explain differential results of previous studies. In Hubbard et al. [10], for example, an italic versus upright discrimination task on the (synaesthesia inducing) graphemes may have enhanced the focus on low-level shape features; effects were predominant in visual areas (V4). In Weiss et al. [14], participants actively reported synaesthetic experiences, enhancing attention to the synaesthetic quality of the colours; effects were found in left parietal cortex only. In our data we found both colour related (V4) and parietal effects, which illustrates that not only task demands, but also the specific aspect of synaesthesia that is under investigation can influence the outcome of neuroimaging experiments.

### Effect of Projector-Associator Type

As the spatial location of the synaesthetic colours can influence experimental outcomes [32], we tested whether the BOLD effects for the synaesthetic condition differed between the projector-associator (PA) subgroups of the synaesthetes (projectors (N = 7), mental screen projectors (N = 7), and associators (N = 5)). The clusters with effects of synaesthesia in right fusiform gyrus and the left superior parietal lobe (SPL) were used as regions of interest (ROIs). Please note that the ROIs were selected completely independent from subgroup status. Mean parameter estimates of each subject (synaesthesia condition) were calculated for each ROI; for neither ROI did we find a main effect of PA-subgroup (ANOVA, all n.s.), although the associator group showed marginally more activity in the left SPL than the mental screen projectors (*F*(1,11) = 3.435, *P*<.094).

## Materials and Methods: Experiment 2

### Priming Experiment

In Experiment 2, we analysed repetition suppression effects for colour induced by synaesthesia, and investigated the effect of projector-associator status on synaesthesia-related activity.

### Participants

All participants of Experiment 1 participated in Experiment 2. Written informed consent was obtained from all participants prior to scanning and the study was approved by the local ethics committee of the Radboud University Nijmegen, in accordance with the Declaration of Helsinki.

### Experimental Design

The priming task contained three synaesthetic priming conditions in which the primes induced a synaesthetic colour that was either congruent (CC) or incongruent (IC) compared to the target, or the primes did not induce a synaesthetic colour (NC). We hypothesised the largest BOLD repetition suppression effects (and hence the lowest BOLD response) would occur for the CC condition. The design also contained real colour priming conditions (CC, IC, and NC), of which the prime and target consisted of physically coloured (or achromatic, not coloured) squares; these trials were intermixed with the synaesthetic priming trials. The colour priming results were reported elsewhere (van Leeuwen, Petersson, Langner, Rijpkema, and Hagoort, submitted).

### Materials

For each synaesthete, 4 graphemes that elicited distinct, vivid synaesthetic colours (mainly red, green, blue, and yellow) were selected from the stimuli of Experiment 1 and used as synaesthesia inducing primes in the CC and IC conditions. Four non-inducing graphemes (no synaesthesia) were selected as primes for the NC condition. For 13 synaesthetes, non-alphanumeric symbols (e.g. &, #) were included as non-inducing primes (2.2 (*SD* = 1.2) on average). Targets consisted of coloured squares in one of the 4 idiosyncratic synaesthetic colours. Control participants received the same stimulus lists as the synaesthete to whom they were matched.

### Stimulus Presentation

All grapheme and symbol stimuli were presented in the same manner as the non-coloured stimuli of Experiment 1. The coloured target squares measured 2.1°×2.1° of visual angle and had a mean luminance of 8.4 cd/m^2^ (*SD* = 10.3 cd/m^2^). Colours were not isoluminant due to their idiosyncratic synaesthetic nature; however, all colours appeared equally often in CC, IC, and NC conditions, ruling out any BOLD effects due to overall luminance differences. Stimuli were presented with the same computer set-up as Experiment 1.

### Procedure

Congruent, incongruent, and non-inducing trials appeared in a ratio of 1∶2∶1 (48∶96∶48), yielding 192 trials (and 192 colour condition trials). The 1∶2∶1 ratio was chosen such that the expectancy of a congruent trial closely matched the expectancy of any target colour (25%), to minimise behavioural strategy effects. Four identical runs were created, each containing 12 CC, 24 IC, and 12 NC trials from both the synaesthetic priming and the colour priming conditions (96 trials per run). Twenty-four null-events (20%, fixation only) were included in each run to avoid BOLD saturation. Stimuli were pseudo-randomised per run, with maximally 2 repetitions of prime type (CC, IC, or NC) and prime identity, maximally 3 repetitions of the target colour, and maximally 5 repetitions of overall condition (synaesthesia or colour).

In the fMRI experiment, one trial consisted of a prime (displayed for 500 ms), followed by a blank screen (100 ms, light grey background colour) and the target (duration 800 ms), and finally a jittered inter-trial-interval of 4–6 seconds (fixation cross). Participants were instructed to indicate the target colour fast but accurately by responding with the associated finger of their right hand; each colour corresponded to one response button. First, participants completed an offline practice set of 16 items (representing all conditions). Response devices were the keyboard for the practice session and an MR-scanner compatible Lumitouch response box for the fMRI experiment. The fMRI session began with the scans from Experiment 1, followed by two runs of the priming experiment (12 minutes each, 380 images). The final 2 priming runs were completed after a 10 minute break outside of the scanner. Participants wore sound-attenuating headphones and two synaesthetes wore additional earplugs to minimise scanner-induced synaesthesias.

### Computer Version

Following the fMRI session, participants completed a computer version of the priming experiment, to verify the behavioural effects obtained in the scanner and to compare the reaction times to the existing literature on synaesthetic priming. Materials and conditions were identical to the fMRI version, but null-events were excluded and the target squares remained on the screen until a response was given (up to a maximum of 4 seconds). Responses were followed by a 1000 ms fixation cross, and then the next trial. Randomisation criteria were unchanged. Four runs of ∼5 minutes each were created. Stimuli were presented on a 15 inch iiyama LCD monitor at a viewing distance of 60 cm. The keyboard was used as response device.

### Image Acquisition Parameters

MR data were collected on the same scanner as the data of Experiment 1. A single shot gradient echo-planar imaging (EPI) sequence was used to acquire functional MR images (33 slices, TE = 30 ms, TR = 2090 ms, flip angle = 80°, 224 mm FOV, 64×64 matrix, 3.5×3.5 mm voxel size, 3.0 mm slice thickness, 0.5 mm slice gap). Atlas-based registration (AutoAlign, Siemens [57]) was applied for all EPI runs to ensure the same slice positions across all functional runs (before and after the break) of one subject. The T1 images from Experiment 1 were used as structural scans.

### Data Analysis

#### Behavioural data

Reaction time (RT) data were analysed in a mixed design ANOVA. Incorrect trials and outliers (±2 *SD* from the subject and condition mean) were excluded from analysis. Where the assumption of non-sphericity was violated, Greenhouse-Geisser correction was applied (uncorrected degrees of freedom are reported).

#### Imaging data

Functional MR data were preprocessed and analysed according to the same procedure as Experiment 1. The design matrix consisted of six regressors for the experimental conditions (CC, IC, and NC in both conditions), one regressor to model all incorrect trials, and six regressors of no interest for the motion parameters. Events were modelled by the onset of the target squares (event related). Analyses were performed at the whole brain level and the initial threshold for significance was a cluster-level statistic of *P*<.05_FWEcorrected_ at the whole brain threshold of *P*<.001_uncorrected_. We used the grapheme and colour volumes of interest (VOIs) from Experiment 1 to aid in the interpretation of the whole brain effects.

Our research question explicitly addressed whether synaesthetic colour perception takes place in *exactly* the same brain areas as real colour perception. We therefore also performed region of interest (ROI) analyses in the same ROIs in which we found RS effects for real colour in our previous study (van Leeuwen, Petersson, Langner, Rijpkema, and Hagoort, submitted). ROI analyses are typically more sensitive than whole brain analyses. The three ROIs were located in left anterior fusiform gyrus, BA 37_priming_ (−32,−50,−22), left posterior fusiform BA 19_priming_ (−30,−66,−22), and right anterior fusiform BA 37_priming_ (32,−50,−24). We included three additional ROIs, on the basis of the local maxima of the colour localiser of Experiment 1 (Table 1: coloured>non-synaesthetic graphemes); these ROIs also showed a main effect of real colour priming across the three conditions, and were located in left anterior fusiform BA 37_localiser_ (−34,−54,−20), and right fusiform gyrus BA 37_localiser_ (34,−50,−22) and BA 19_localiser_ (32,−76,−16). ROIs (5 mm radius) were created with MarsBaR [47]. For each ROI, the mean parameter estimates for each subject and condition were extracted and subjected to statistical analysis.

## Results: Experiment 2

One participant was excluded from analysis of Experiment 2 because she did not complete the task according to instructions. The remaining 19 synaesthetes and their 19 matched controls were included.

### Behavioural Results

**Figure 2:**
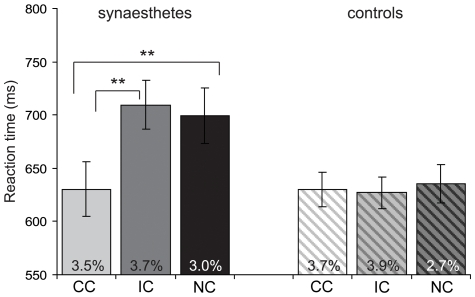
Reaction time effects of synaesthetic priming. RTs for the congruent (CC), incongruent (IC), and non-inducing (NC) conditions of the synaesthetic priming experiment, for synaesthetes (solid bars, N = 19) and controls (shaded bars, N = 19). Data are collapsed over fMRI and computer sessions. Error percentages are listed for each condition and ± error bars denote the standard error of the mean. ** (*P*<.001) indicate significant effects.

RTs were analysed in an ANOVA with the between-subjects factor group (synaesthetes and controls), and the within-subjects factors place (fMRI and computer) and prime type (congruent, incongruent, and non-inducing). Incorrect responses (for percentages see Figure 2) and outliers (fMRI: synaesthetes 5.6%, controls 5.8%; computer: synaesthetes 4.6%, controls 4.7%) were removed prior to analysis. We found a main effect of place (*F*(1,36) = 27.8, *P*<.001), caused by longer RTs in the scanner, but no main effect of group (*F*(1,36) = 2.33, n.s.), thus the overall RTs were comparable across groups. Because no interactions with place were found for either group, data were collapsed across fMRI and computer sessions.

The collapsed data revealed a highly significant group×prime type interaction (*F*(2,148) = 23.9, *P*<.001). We found a significant effect of prime type for synaesthetes (*F*(2,74) = 27.5, *P*<.001), but not for controls (*F*(2,74) = 1.83, n.s.). For synaesthetes the RTs in the congruent colour condition were 79 ms faster than those in the incongruent colour condition (*F*(1,37) = 32.3, *P*<.001), and 69 ms faster than in the non-inducing condition (*F*(1,37) = 33.2, *P*<.001). There were no other effects; the data are summarised in Figure 2. The pattern of RT effects indicates interference effects for synaesthetes for the IC and NC trials, comparable to previously reported reaction time effects for synaesthesia [25], [26].

The error rates were analysed to check for possible speed-accuracy trade-offs. A prime type×group ANOVA revealed an effect of prime type (*F*(2,148) = 4.23, *P*<.05): separate group analyses revealed an effect of prime type for the controls only (*F*(2,74) = 3.56, *P*<.05; synaesthetes *F*(2,74) = 1.06, n.s.). The controls made slightly more errors in the IC condition than in the NC condition: *F*(1,37) = 6.13, *P*<.05. The increased IC error rate suggests that the control subjects were learning the correct (congruent) prime-target associations during the experiment.

### fMRI Results

#### Whole brain analyses

We first ascertained that there were no overall differences between synaesthetes and controls; a group×prime type factorial model revealed no main effect of group (at *P*<.001_uncorrected_). It is therefore unlikely that synaesthetes and controls made use of different strategies or that task difficulty affected the groups differently. Next, we tested whether the presence of synaesthesia-inducing primes (in congruent synaesthetic colour and incongruent synaesthetic colour conditions) would lead to a general colour repetition suppression effect compared to the non-inducing primes (NC condition), for synaesthetes. Because repetition suppression would lower the BOLD response for CC and IC conditions, such an effect would lead to relatively higher BOLD activity for the NC condition compared to the combined CC and IC conditions. The interaction and main effects that we found for synaesthetes for this comparison (see Table 2) were not located within the pre-defined colour VOI but in the grapheme VOI; hence we cannot claim that synaesthesia induces repetition suppression for *colour*. The stronger activation for NC trials may be explained by the relatively infrequent occurrence of the individual non-inducing NC primes (individual NC primes appeared 12 times (48/4 NC), individual synaesthesia inducing primes 36 times (48/4 CC+96/4 IC)). No effects were found for the controls.

**Table 2:** Synaesthetic priming effects in the brain.

Contrast	Brain region	k	p	MNI (x,y,z)	T-value
**Interaction Synaesthetes>Controls for NC>CC&IC**	R Superior frontal gryus (BA 9)	270	0.061	20, 34, 38	4.46
	R Superior frontal gryus (BA 9)			20, 42, 34	3.86
	L Superior frontal gyrus (BA 9/10)	386	0.019	−22, 42, 32	4.42
	L Middle frontal gyrus (BA 9/8)			−26, 28, 40	3.71
**SVC: NC>(CC & IC) for Synaesthetes (grapheme VOI)**	R Inferior temporal gyrus (BA 37/19)	257	0.002	48, −72, −8	4.51
	R Fusiform/Inferior temporal gyrus (BA 37/20)			48, −56, −18	4.18
	R Inferior temporal gyrus (BA 37/20)			48, −54, −22	4.08
	L Fusiform/Inferior occipital gyrus (BA 37/19)	41	0.040	−44, −62, −12	3.75
**IC>CC for Synaesthetes**	R Superior frontal gyrus (BA 6)	886	0.000	26, 0, 52	4.70
	R Precentral gyrus (BA 4/6)			44, −20, 58	4.08
	R Postcentral gyrus (BA 4/3)			30, −32, 58	3.86
	R Superior temporal gyrus (BA 21)	493	0.007	56, −6, −14	4.27
	R Middle temporal gyrus (BA 21)			56, 2, −18	4.24
	R Hippocampus			38, −12, −24	4.17

fMRI results of synaesthetic priming Experiment 2 (N = 19 synaesthetes, N = 19 controls). Random effects analyses, extent threshold ≥50 voxels, whole brain threshold *P*<.001_uncorrected_. Cluster size (k), corrected P-values at cluster-level (p), MNI coordinates of the local maxima and T-values are listed. Brodmann areas (BA) are in parentheses. R = right, L = left, NC = non-inducing condition, CC = congruent synaesthetic colour condition, IC = incongruent synaesthetic colour condition, SVC = Small Volume Correction, VOI = Volume of Interest.

**Figure 3:**
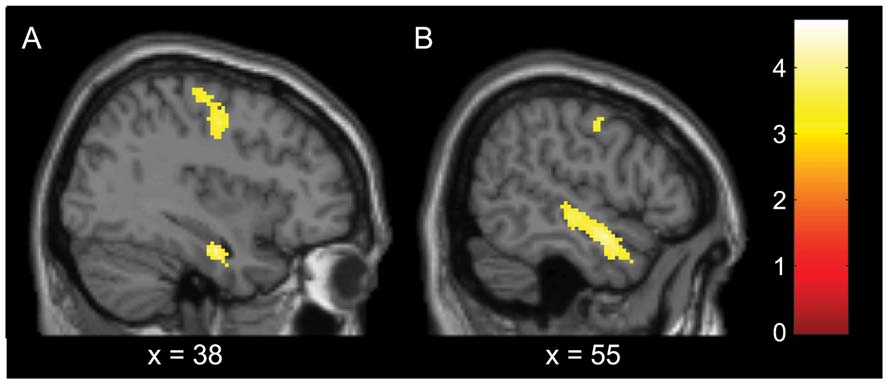
Brain areas with repetition suppression effects for synaesthetic colour. fMRI results of synaesthetic priming Experiment 2, showing whole brain effects of the incongruent synaesthetic colour condition compared to the congruent synaesthetic colour condition, reflecting repetition suppression effects in the congruent colour condition (for synaesthetes only, N = 19, whole brain *P*<.001_uncorrected_). Clusters significant at cluster-level *P*<.05_FWEcorrected_ are shown (see Table 2). Sagittal slices through the right hemisphere show effects in A. right superior frontal gyrus and right hippocampus, and B. right middle/superior temporal gyrus. Anterior is depicted on the right. The legend denotes T-values.

Our main hypothesis stated that the CC condition would induce more repetition suppression in synaesthetes than the IC condition, due to the neuronal overlap in colour processing between the synaesthetic prime colour and the real colour target. More repetition suppression in the CC condition would mean that the BOLD response to the CC condition would be reduced more than the BOLD response to the IC condition. Hence the IC condition would lead to more BOLD activity than the CC condition, in case our hypothesis were true. The comparison of interest was therefore between IC and CC conditions. No interaction effects between synaesthetes and controls were found, but for synaesthetes only there were significant differences (at *P*<.001_uncorrected_) in the right superior frontal gyrus (BA 6) and a cluster of activation in the right temporal gyrus (BA 21), including a local maximum in the hippocampus (Table 2 and Figure 3). These clusters, however, were not located in the colour VOI or the grapheme VOI, and therefore we did not interpret them as repetition suppression effects related to colour. There were no significant effects for the controls.

Taken together, the whole brain analyses provide no evidence that synaesthetically induced colour leads to repetition suppression effects for real colour perception in any colour sensitive areas. We now turn to more sensitive analyses, within ROIs in which repetition suppression effects have been found for real colour priming; we compare the synaesthetic effects to the real colour effects.

#### Region of interest analyses

No significant group×prime type interaction, nor main effect of group were found in any of the 6 ROIs that we tested, but main effects of prime type were present in four ROIs. These were located in left anterior fusiform gyrus (BA 37_priming_: *F*(2,72) = 6.08, *P*<.01 and BA 37_localiser_: *F*(2,72) = 7.81, *P*<.001) and right anterior fusiform gyrus (BA 37_priming_: *F*(2,72) = 3.39, *P*<.05 and BA 37_localiser_: *F*(2,72) = 4.83, *P*<.05). Because we hypothesised differential effects would occur for synaesthetes and controls the data were split by group. Surprisingly, there were no effects of prime type for the synaesthetes in any ROI, indicating that the synaesthetic priming manipulation did not affect processing of real colour for synaesthetes.

**Figure 4:**
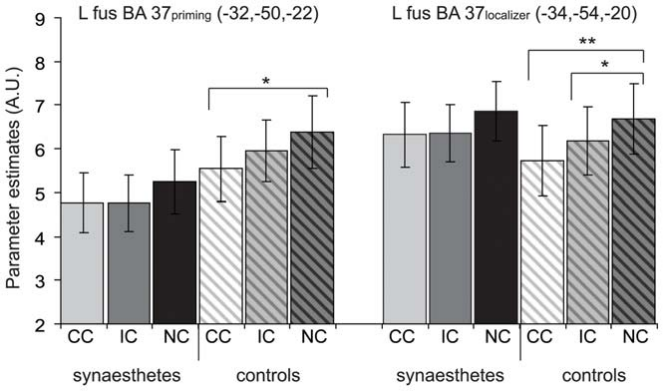
Region of interest analysis of repetition suppression effects for synaesthetic priming. The mean parameter estimates for ROIs in left anterior fusiform gyrus are plotted for synaesthetes (N = 19, solid bars) and controls (N = 19, shaded bars). Error bars depict ± the standard error of the mean and * (*P*<.05) and ** (*P*<.001) denote significant differences between conditions. CC = congruent synaesthetic colour condition, IC = incongruent synaesthetic colour condition, NC = non-inducing condition, L = left, fus = fusiform gyrus.

In two ROIs in the left anterior fusiform gyrus we encountered an unpredicted main effect of prime type for the control participants (BA 37_priming_: (*F*(2,36) = 7.24, *P*<.01 and BA 37_localiser_: (*F*(2,36) = 9.53, *P*<.001). Effects for the controls were driven by increased BOLD responses for the NC condition (see Figure 4), most likely due to the relatively infrequent occurrence of the individual primes in the NC condition.

In addition to regular ROI analyses, we also created individual ROIs with maximal colour sensitivity for each subject, to increase sensitivity. On the basis of the contrast of coloured graphemes compared to non-inducing control graphemes from Experiment 1, we successfully determined two ROIs (5 mm radius, one left lateralised, one right lateralised) in visual cortex for seventeen subjects from each group. An effect of prime type for the real colour conditions was found only in left lateralised ROIs (F(2,64) = 3.84, P<.05), and we therefore only looked for effects of synaesthetic priming in the left lateralised ROIs as well. A main effect of the synaesthesia prime type manipulations in the left ROIs (F(2,64) = 5.23, P<.05) was driven by the controls (F(2,32) = 3.53, P<.05), due to enhanced activity for the NC condition compared to the CC condition (F(1,16) = 4.50, P<.05).

### Main Effect of Synaesthesia

In the priming experiment, synaesthesia was elicited in the CC and IC conditions, and not in the NC condition: we therefore investigated whether the CC and IC conditions would lead to similar main effects of synaesthesia as those that were found in Experiment 1. No significant positive interactions were found for synaesthetes compared to control subjects, for the contrast of the combined CC and IC conditions compared to the NC condition (at whole brain *P*<.001_uncorrected_).

As mentioned previously, the experiment also contained a colour priming version of the synaesthetic priming task (van Leeuwen, Petersson, Langner, Rijpkema, and Hagoort, submitted), with the same manipulations (CC, IC, and NC) and task. Here, we used the colour priming conditions as a baseline and looked for the added effect of synaesthetic colour experience for synaesthetes, compared to control participants. The comparison with control participants makes sure that effects of the stimuli themselves, expected for both groups, are cancelled out. In the left superior parietal lobe (SPL), a marginally significant (at whole brain *P*<.001_uncorrected_) positive interaction for synaesthetes was found for the contrast of the collapsed synaesthesia CC and IC conditions, compared to the collapsed colour CC and IC conditions (see Table 3). The effect was near the left SPL synaesthesia cluster that we reported in localiser Experiment 1, and became highly significant when we restricted the analysis (SVC) to a 15 mm radius sphere around the SPL localiser cluster (Table 3). The results emphasise the importance of the left superior parietal lobule for the experience of synaesthetic colour.

**Table 3:** Main effects of synaesthesia in the synaesthetic priming experiment.

Contrast	Brain region	k	p	MNI (x,y,z)	T-value
**Interaction Synaesthetes>Controls for Synaesthetic (CC & IC)>Colour (CC & IC)**	L Superior parietal lobule/Prec (BA 7)	252	0.077	−26, −60, 38	4.16
	L Superior parietal lobule (BA 7)			−30, −54, 56	3.47
**SVC: 15 mm sphere at SPL (−24, −58, 46)**	L Superior parietal lobule/Prec (BA 7)	238	0.002	−26, 60, 38	4.16
	L Superior parietal lobule (BA 7)			−26, −60, 44	3.95
	L Superior parietal lobule (BA 7)			−30, −54, 56	3.47

fMRI results for the interaction of synaesthetes compared to controls, for the synaesthetic CC and IC conditions compared to the real colour CC and IC conditions (N = 19 synaesthetes, N = 19 controls) of Experiment 2. Random effects analyses, extent threshold ≥50 voxels, whole brain threshold *P*<.001_uncorrected_. Listed are cluster size (k), corrected P-values at cluster-level (p), MNI coordinates of local maxima and T-values. Brodmann areas (BA) are in parentheses. R = right, L = left, CC = congruent (synaesthetic) colour condition, IC = incongruent (synaesthetic) colour condition, SVC = Small Volume Correction, Prec = Precuneus.

### Effect of Projector-Associator Type

**Figure 5:**
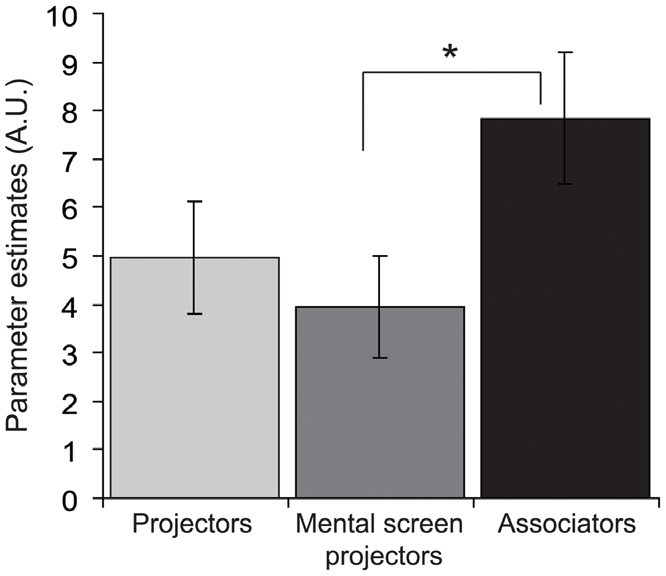
Projector-associator differences in the superior parietal lobe. Mean parameter estimates (Experiment 2) for projector, mental screen projector, and associator synaesthetes in the left superior parietal lobule cluster at (−26, −60, 38) (Table 2). Error bars depict ± standard error of the mean. * (*P*<.05) denotes significant difference between groups.

We tested whether the effect of synaesthesia in the left SPL synaesthesia cluster was different for the three projector-associator (PA) subgroups of the synaesthetes (projectors (N = 7), mental screen projectors (N = 7), and associators (N = 5)). We calculated the mean parameter estimates of the CC and IC conditions for each subgroup, for the voxels belonging to the left SPL cluster with an effect of synaesthesia in the priming experiment (Table 3). No other ROIs were tested because we constrained our analyses to areas that were positively involved in synaesthesia (but selected independent from subgroup status). A marginal main effect of PA-subgroup was found: *F*(2,37) = 2.60, *P*<.09. Planned comparisons revealed that the associators showed significantly more activation in the left SPL than the mental screen projectors (*F*(1,23) = 5.29, *P*<.05), see Figure 5. This finding is comparable to the trend that was found in the left SPL cluster in the localiser experiment.

## Discussion—General

The aim of our study was to determine whether synaesthetically induced colour perception recruits the same brain areas as real colour perception. Although we know from neuroimaging studies that ventral-occipital colour areas are involved in synaesthetic experiences (e.g.,[9], [10]), to our knowledge it has not been shown that synaesthetically induced colours can affect real colour processing in the brain. Such an influence would imply the neural machinery is truly shared between synaesthetic and real colours. In our synaesthetic priming experiment we applied a sensitive, repetition suppression fMRI paradigm; we hypothesised that synaesthetic colours would reduce the subsequent BOLD response for real colours. We found no effects, and therefore no evidence for shared neural correlates between synaesthetically induced colour and real colour perception.

Behaviourally, the reaction time effects we obtained for synaesthetic priming were of the same order of magnitude (colour: 56 ms, synaesthesia: 79 ms) as previously established colour priming effects in the same subjects (van Leeuwen, Petersson, Langner, Rijpkema, and Hagoort, submitted). It follows that the physical colours of our target stimuli were matched closely enough to the idiosyncratic prime colours induced by synaesthesia to elicit strong behavioural interference. In the brain however, real colour priming induced repetition suppression effects in visual area V4α (van Leeuwen, Petersson, Langner, Rijpkema, and Hagoort, submitted), whereas we did not find any effects for synaesthetic priming, tested with the same number of stimuli and identical task. For synaesthetic priming we performed additional region of interest analyses, but to no avail. In our free viewing localiser experiment (Experiment 1) we did find an effect of synaesthesia in colour area V4. This indicates that V4 was positively involved in synaesthesia in our synaesthetes, and repetition suppression effects could in principle have been induced in the priming experiment. The number of stimuli in the localiser experiment and priming experiment were comparable; a difference in experimental power can therefore not explain the absence of effects in the priming study. The effect that we found for the non-inducing condition in control subjects (due only to frequency effects of the stimuli) also makes a lack of sensitivity unlikely. We therefore accept that synaesthetic colour processing indeed does not influence real colour processing in ventral-occipital areas in the brain.

In the reaction times, we found a priming effect for the congruent synaesthetic colour condition, in accordance with previous findings for synaesthetic priming (e.g., [26]). The reaction times were faster when the synaesthetically induced colour of the prime matches with the colour of the target. The fact that we did not find repetition suppression effects in visual cortex for this comparison, suggests that the conflict that is induced by the non-matching colours in the incongruent condition is resolved elsewhere in the brain. In this sense, our fMRI data complement earlier behavioural studies that have only assessed reaction times without looking at the neural correlates. In the contrast of the incongruent versus the congruent condition in the brain (see Table 2) we observed activations in right superior frontal gyrus (motor-related areas) and right temporal gyrus, areas in which this conflict is possibly resolved. The finding that repetition suppression for stimulus features in sensory cortex can be independent from observable measures like reaction times and response learning has previously been demonstrated by Horner and Henson [38].

In our localiser experiment (Exp. 1), we found increased BOLD activity for synaesthetic colour perception in the fusiform gyrus and increased activity for synaesthetic colour binding processes in left parietal cortex. These results are in line with the proposed neural correlates of synaesthesia in the integrated model put forward by Hubbard [19], [20]. We can infer at which level of visual processing the neural correlates of synaesthetically induced colour could be encountered. Although behavioural studies show that synaesthetically induced colours can ‘behave’ like real colour (e.g., [31]), many of these studies were case studies, including participants who exhibited very strong, low-level synaesthesia (e.g., [4]). This may have resulted in potential overestimation of the perceptual effects of synaesthesia, and therefore of the earliest level of visual processing at which neural correlates of synaesthesia can be identified. In our study, the most low-level neural correlate of synaesthesia that we encountered was extrastriate visual area V4. Even in V4 and V4α, synaesthetically induced colours did not affect BOLD activity in the same way real colours did, implying that the neural machinery that is underlying synaesthetic colour experience is not organised in the same way as the neural machinery underlying real colour perception. Our results suggest that synaesthetic colours are mediated by higher-order (visual) processes, taking place beyond the realm of well-defined visual areas in ventral-occipital cortex. Feedback from these areas may induce V4 activation and the percept of synaesthetic colour, and the left superior parietal lobe most likely has an important role in this process. We propose that the pathways by which synaesthetically induced colour and real colour are processed by the brain are different in nature, even though both may result in V4 activation. Studies of functional and effective connectivity, in which the dynamic interplay between brain areas is modelled, may help to advance our understanding of the nature of the connections between brain areas that are involved in synaesthetic colour experiences. Electroencephalography (EEG) and magnetoencephalography (MEG) may provide valuable information about the time course with which synaesthesia-inducing stimuli are processed, considering that these methods have a very high time resolution. If it is possible to spatially localise the sources of EEG and MEG activity, these methods may also assist in determining the order in which brain regions are recruited during the experience of synaesthesia.

Several of our synaesthetes experienced the colours induced by synaesthesia in a specific spatial location (projectors), whereas for others they resembled strong associations (associators). In the left superior parietal lobule (SPL), we found significantly more activity for associator synaesthetes than for mental screen projectors. This finding is unexpected if one interpreted the spatial component of synaesthesia as reflecting the underlying binding processes to spatial reference frames [21], [23], [24]; for projector synaesthetes, the spatial reference frame of the colour is more explicit and hence may lead to increased BOLD responses. Alternatively, if associator synaesthetes build a spatial reference frame for their synaesthetic colours that differs largely from the way graphemes are presented on the experimental computer screen, the switching between spatial reference frames and increased difficulty in spatial binding may lead to increased SPL activity [21]. Our data do imply that activity in the left superior parietal lobe is related to these individual differences.

In summary, our data support an integrated model of synaesthesia, and suggest the specific aspect of synaesthesia that is under investigation (colour perception or the specific synaesthetic aspect of the colour) can influence experimental outcomes. The left superior parietal cortex is implied in the spatial reference frame of synaesthesia. Synaesthetically induced colours do not coincide with real colour perception in the brain, which suggests that synaesthetic colour perception is mediated in higher-order (visual) areas. Feedback, caused by either anatomical or functional connectivity, may induce activation of visual area V4. In the future, functional and effective connectivity methods and models may further elucidate the neural underpinnings of synaesthetic colour experiences.
